# Endogenous and Exogenous KdpF Peptide Increases Susceptibility of *Mycobacterium bovis* BCG to Nitrosative Stress and Reduces Intramacrophage Replication

**DOI:** 10.3389/fcimb.2017.00115

**Published:** 2017-04-06

**Authors:** Mariana Rosas Olvera, Eric Vivès, Virginie Molle, Anne-Béatrice Blanc-Potard, Laila Gannoun-Zaki

**Affiliations:** ^1^Laboratoire de Dynamique des Interactions Membranaires Normales et Pathologiques, Université MontpellierMontpellier, France; ^2^Centre National de la Recherche Scientifique, UMR5235Montpellier, France; ^3^CRBM, Centre National de la Recherche Scientifique UMR 5237Montpellier, France

**Keywords:** anti-virulence strategy, *Mycobacterium bovis* BCG, KdpF, membrane peptide, nitrosative stress, macrophage, protein-protein interactions

## Abstract

Emerging antibiotic resistance in pathogenic bacteria like *Mycobacterium* sp., poses a threat to human health and therefore calls for the development of novel antibacterial strategies. We have recently discovered that bacterial membrane peptides, such as KdpF, possess anti-virulence properties when overproduced in pathogenic bacterial species. Overproduction of the KdpF peptide in *Mycobacterium bovis* BCG decreased bacterial replication within macrophages, without presenting antibacterial activity. We propose that KdpF functions as a regulatory molecule and interferes with bacterial virulence, potentially through interaction with the PDIM transporter MmpL7. We demonstrate here that KdpF overproduction in *M. bovis* BCG, increased bacterial susceptibility to nitrosative stress and thereby was responsible for lower replication rate within macrophages. Moreover, in a bacterial two-hybrid system, KdpF was able to interact not only with MmpL7 but also with two membrane proteins involved in nitrosative stress detoxification (NarI and NarK2), and a membrane protein of unknown function that is highly induced upon nitrosative stress (Rv2617c). Interestingly, we showed that the exogenous addition of KdpF synthetic peptide could affect the stability of proteins that interact with this peptide. Finally, the exogenous KdpF peptide presented similar biological effects as the endogenously expressed peptide including nitrosative stress susceptibility and reduced intramacrophage replication rate for *M. bovis* BCG. Taken together, our results establish a link between high levels of KdpF and nitrosative stress susceptibility to further highlight KdpF as a potent molecule with anti-virulence properties.

## Introduction

Recent efforts in genomics and natural product discovery led to a tremendous increase in the number and diversity of very small proteins (size below 50 amino-acids, called hereafter peptides) produced by bacteria (Flaherty et al., 2014). A few studies have highlighted the regulatory role of bacterial membrane peptides, which harbor a single transmembrane domain (Alix and Blanc-Potard, 2009; Storz et al., 2014). These peptides seem to play a regulatory role by interacting with protein partners localized at the membrane, thereby modulating their activity and/or stability. Interestingly, some peptides as KdpF and MgtR, were shown to modulate bacterial virulence and have been proposed as attractive anti-virulence peptides (Gannoun-Zaki et al., 2013; Belon et al., 2016).

We have recently demonstrated that a constitutive overproduction of the 30 amino-acid KdpF membrane peptide in *Mycobacterium bovis* BCG resulted in altered cording morphology and reduced intramacrophage growth (Gannoun-Zaki et al., 2013). The Kdp system, which has been extensively studied in *Escherichia coli*, is a P-type ATPase transporting K^+^ ions with high affinity (Greie and Altendorf, 2007; Greie, 2011). The Kdp system consists of four subunits, which are encoded in the *kdpFABC* operon. KdpA subunit provides the K^+^ binding site and the K^+^ translocation channel whereas KdpB subunit is in charge of the K^+^ uptake and KdpC is required to stabilize the complex (Heitkamp et al., 2009; Irzik et al., 2011). Upon K^+^ limitation, *kdpFABC* operon expression is activated by the two-component system KdpD/KdpE. Under these conditions, the histidine kinase KdpD autophosphorylates and transfers the phosphoryl group to the response regulator KdpE. Once phosphorylated, KdpE exhibits higher affinity for the *kdpFABC* promoter region to trigger transcription (Ballal et al., 2007; Heermann and Jung, 2010). In *E. coli*, the KdpF peptide was proposed to be a subunit involved in the stabilization of the KdpABC complex *in vitro*, although its role *in vivo* remains elusive as it is not essential for the function of the Kdp transporter (Gässel et al., 1999). In our previous work (Gannoun-Zaki et al., 2013) we have shown that the *M. bovis* BCG *kdp* operon is induced under K^+^ limitation and in macrophages, but overexpression of *kdpF* did not alter the expression pattern of *kdp* genes, indicating that KdpF does not play a regulatory role on the KdpD kinase. On the other hand, our previous results indicated that the KdpF peptide was able to interact with MmpL7, involved in the transport of cell wall phthiocerol dimycoserates lipids (PDIM) (Cox et al., 1999; Bailo et al., 2015), highlighting a possible link between KdpF and cell wall lipid metabolism (Gannoun-Zaki et al., 2013). We thus hypothesized that the reduced intramacrophage growth upon KdpF overproduction may result from alteration of the mycobacterial cell wall. We showed that KdpF overproduction did not increase membrane permeability, but whether KdpF overproduction could increase the susceptibility to stresses encountered by the bacteria inside macrophages remains to be investigated.

Nitrosative stress is one of the stresses encountered by mycobacteria within macrophages and production of nitric oxide (NO) by infected macrophages is one of the defense mechanism against *M. tuberculosis* (Ehrt and Schnappinger, 2009). *M. tuberculosis* has developed various strategies to overcome killing by macrophages, including alterations in production of the superoxide radicals, such as NO (Kumar et al., 2011). Several proteins of the Nar complex are known to be involved in the nitrosative stress response in *M. tuberculosis* (Gouzy et al., 2014). The inorganic nitrogen source, nitrate (${\text{NO}}_{3}^{-}$), is imported into the bacteria through the NarK2 membrane protein. Then, nitrates are reduced to nitrites (${\text{NO}}_{2}^{-}$) by the nitrate reductase complex proteins (NarGHIJ) (Huang et al., 2015). The resulting nitrites are further reduced to nontoxic ammonium ions (${\text{NH}}_{4}^{+}$) by the cytoplasmic nitrite reductase complex NirB-NirD, or secreted by putative nitrite extrusion proteins (Gouzy et al., 2014; Huang et al., 2015). The membrane-bound nitrate reductase complex consists of NarG, NarH, and NarI, with NarG bearing the catalytic subunit, whereas NarJ is required for the assembly of NarGH prior to its attachment to NarI in the membrane. NarI subunit is completely immersed in the membrane and is associated with the NarGH dimer through a hydrophobic patch present in NarH (González et al., 2006). In addition, some genes, such as *Rv2617c*, encoding a membrane protein of unknown function, have been shown to be highly upregulated upon nitrosative stress in *M. tuberculosis* (Ohno et al., 2003). On the other hand, genes involved in PDIM synthesis were strongly down-regulated upon nitrosative stress in *M. tuberculosis* (Cossu et al., 2013), indicating that these lipids may be turned off by *M. tuberculosis* in the presence of reactive nitrogen species.

In the present work, we demonstrated that KdpF overproduction increased the bacterial susceptibility to nitrosative stress and that this susceptibility is likely to be responsible for the defect in intramacrophage replication. We thus studied a potential regulatory effect of KdpF on membrane proteins involved in nitrosative stress response. Our work revealed that KdpF could interact with such proteins and may modulate their stability, thus providing a potential mechanistic clue explaining the nitrosative stress susceptibility. Finally, we showed that exogenous addition of KdpF synthetic peptide could mimic the phenotype obtained upon KdpF endogenous overproduction, thus confirming the prospective of KdpF as an anti-virulence molecule.

## Materials and methods

### Bacterial strains and growth conditions

*Mycobacterium bovis* BCG (Pasteur 1173P2) overproducing the KdpF peptide (pKdpF) and its control strain (containing the empty vector, pMV261) were used in this study (Gannoun-Zaki et al., 2013). The overexpression of *kdpF* gene from the pMV261 vector (Stover et al., 1991) is triggered by the *hsp60* promoter and is therefore constitutive. Both strains were grown at 37°C in liquid culture in Sauton's medium containing 0.025% tyloxapol (Sigma), in the presence of kanamycin (25 μg/ml) as required or onto Middlebrook 7H10 agar (Difco) plates supplemented with Oleic Acid-Dextrose-Catalase (OADC). Plates were incubated at 37°C for 2–3 weeks prior to visual counting of the colony forming units (CFUs).

### Synthesis of a KdpF peptide derivative

Peptide synthesis was performed by a solid-phase method using the Fmoc methodology on an automated microwave peptide synthesizer (Liberty-1, CEM, Orsay, France) as described previously (Belon et al., 2016). Due to failure in the synthesis of the entire KdpF peptide (MTTVDNIVGLVIAVALMAFLFAALLFPEKF), a shorter peptide deleted for 5 amino-acids at the N-terminus (MIVGLVIAVALMAFLFAALLFPEKF) was synthesized. The final product was resuspended at 3.5 mM in DMSO/water (25%/75%, vol/vol) and analyzed by MALDI-TOF mass spectrometry.

### Macrophage culture and infection

The human monocytic cell line THP-1 was obtained from the ATCC collection (TIB-202™). Cells were grown at 37°C in a 5% CO_2_ atmosphere in RPMI 1640 with Glutamax I (Life Technology) supplemented with 10% fetal calf serum, 0.1 mM non-essential amino acids and 0.15% sodium pyruvate (Life Technology). Cultures were maintained at a density not > 5 × 10^5^ cells/ml.

For infection experiments, THP-1 cell suspensions were adjusted to a concentration of 2 × 10^5^ cells/ml in complete RPMI medium, seeded in 24-well plates, and cells were allowed to adhere and differentiate in the presence of 60 nM Phorbol 12-Myristate 13-Acetate (PMA, Sigma) for 72 h at 37°C. The medium of differentiated THP-1 cells was removed and cells were infected with exponentially growing *M. bovis* BCG cultures (OD_600_ = 0.8) at a multiplicity of infection (MOI) of 5:1 bacteria per macrophage as previously described (Corrales et al., 2012). After 3 h of incubation, the infected cells were washed 3 times with PBS buffer to remove any extracellular bacteria, and then incubated in fresh medium for 6 days. For scoring, cells were lysed with 0.1% Triton X-100 in PBS at selected times post-infection and serial dilutions of the lysate were plated onto agar plates.

To inhibit reactive nitrogen intermediates (RNIs) production, 200 μM N-ω-nitro-L-arginine methyl ester hydrochloride (L-NAME, Sigma) was added to the culture medium of activated macrophages 1 h before infection and maintained throughout the infection process. Data were normalized to multiplication of the *M. bovis* BCG pMV261 strain in non-treated L-NAME macrophages to account for differences in bacterial multiplication between the two bacterial strains in macrophages L-NAME-treated or not.

To test the effect of synthetic KdpF on intramacrophage bacterial replication, *M. bovis* BCG pMV261 was incubated with synthetic KdpF (70 μM) during 10 min at room temperature. These pre-treated bacteria were used to infect THP-1 macrophages and plated after 3 h and 6 days post infection onto agar plates.

### Determination of mycobacterial susceptibility to exogenous NO

*Mycobacterium bovis* BCG pKdpF and *M. bovis* BCG pMV261 were grown to mid log phase and diluted to obtain 3 × 10^7^ bacteria/ml. Tubes containing the bacilli were incubated for 5 h at 37°C in Sauton's medium containing 0.1 mM or 1 mM of the NO donor SNAP (S-nitroso-N-acetyl-DL-penicillamine, Sigma) (Ferrero et al., 1999) supplemented with 10 mM CuSO_4_ (Nelli et al., 2000). Serial dilutions were plated onto Middlebrook 7H10 agar containing 25 μg/ml kanamycin.

To test the effect of synthetic KdpF peptide on mycobacterial susceptibility to NO stress, tubes containing *M. bovis* BCG pMV261 (1.5 × 10^7^ bacteria) were incubated at 37°C for 5 h in Sauton's medium containing 70 μM of synthetic KdpF and 1 mM of the NO donor SNAP, supplemented with 10 mM CuSO_4_. Serial dilutions were plated as described earlier. The obtained CFUs from the control cultures (also supplemented with 10 mM CuSO_4_-DMSO 25%) were considered as 100% viability.

### Quantification of NO production by infected THP-1 cells

THP-1 cells were infected with *M. bovis* BCG pMV261 and *M. bovis* BCG pKdpF as described earlier. Supernatants of infected THP-1 cells were collected after 3 h and 6 days. The production of NO was indirectly measured by assaying for the presence of nitrite (${\text{NO}}_{2}^{-}$) using the Griess reagent (Green et al., 1982). Aliquots of 100 μl of culture media were distributed in 96-well microtiter plates and equal volumes of Griess reagent solution were added. The reaction was allowed to proceed for 15 min at room temperature, and the optical density at 540 nm was measured. The amounts of produced NO were quantified from a standard curve using dilutions of NaNO_2_ (100 nM–100 μM).

### RNA extraction and quantitative RT-PCR (qRT-PCR)

RNA was prepared as previously described (Gannoun-Zaki et al., 2013) from 5 ml of mid-logarithmic bacterial cultures grown in Sauton's medium supplemented with 10 mM CuSO_4_ or grown for 5 h in Sauton's medium supplemented with 1 mM SNAP. Quantitative real-time PCR was performed using an in-house SYBR Green mix (Lutfalla and Uze, 2006) and a 480 light cycler instrument (Roche). PCR conditions were as follows: 3 min denaturation at 98°C, 45 cycles of 98°C for 5 s, 68°C for 10 s, and 72°C for 10 s. The 16S ribosomal RNA gene (*rrs*) was used as internal control to calculate the relative level of gene expression. The sequences of primers for qRT-PCR are listed in Table 1.

**Table 1:** **Primers used in this study for qRT-PCR and for cloning into pUT18 vector (-T18)**.

**Primer**	**Sequence**
16S-BCG-F	5′-TGTGAGCGTGCGGGCGATAC
16S-BCG-R	5′-ACGGCGTGGACTACCAGGGT
NarI-BCG-F	5′-ATGTACCTGGTGCTGGTGTG
NarI-BCG-R	5′-ATGCACCTGGTAGTACAGCG
NarK2-BCG-F	5′-TGGTGGCCATGGTCGTGCTT
NarK2-BCG-R	5′-GCCGAACACGATCGCGTACA
MmpL7-BCG-F	5′-TGGAAACGATCCACACCTGG
MmpL7-BCG-R	5′-TGTCTGTGTCGATATCGCGG
Rv2617c-BCG-F	5′-GAGCATCAGACCAACGACCA
Rv2617c-BCG-R	5′-ACGAGATCGTTGATCCAGCC
Del-KdpF-BCG-F	5′-ATCGTCGGGTTGGTGATCGC
Del-KdpF-BCG-R	5′-CATGGGATCCTCTAGAGTCG
NarI-HindIII-T18	5′-CCCAAGCTTGTTGGCCGTCTTGGACTTGGTTG
NarI-XbaI-T18	5′-GGGTCTAGAGTCCACCCACGACGACGCGGCGCG
Nark2-HindIII-T18	5′-C CCAAGCTTGATGAGAGGGCAAGCGGCCAAT
Nark2-XbaI-T18	5′-GGGTCTAGAGTCCTGGACGCCTCCTCACTCAC
SecG-Hind-T18	5′-CCCAAGCTTGATGGAATTGGCCCTGCAGATCAC
SecG-Bam-T18	5′-CGGGATCCTCGCGGTATTTGATGAGCAACGCC

*F and R stand for forward and reverse, respectively*.

The comparison of gene expression level in *M. bovis* BCG strains cultured in presence or not of 1 mM SNAP was performed with REST 2009 software (Vandesompele et al., 2002) and plotted using GraphPad-Prism software.

### Bacterial two-hybrid analysis

The Bacterial Adenylate Cyclase Two-Hybrid (BACTH) system (Karimova et al., 1998) was used to evaluate the interaction between KdpF and MmpL7, NarI, NarK2, Rv2617c, or SecG. The KdpF-T25 and MmpL7-T18 constructions were previously described (Gannoun-Zaki et al., 2013). To generate the pUT18-NarI, pUT18-NarK2, and pUT18-SecG constructions (plasmids are listed in Table 2), genes were PCR amplified from *M. tuberculosis* genomic DNA using the primers listed in Table 1. The plasmid pUT18-Rv2617c was provided by Dr. Fabiana Bigi (Klepp et al., 2009). Recombinant plasmids derived from pUT18 and pKT25 genes were co-transformed into *E. coli* BTH101 bacteria and BACTH assay were conducted at 30°C as described (Karimova et al., 1998). A level of β-galactosidase activity at least 5-fold higher than the one of the control vectors indicates an interaction between the protein partners.

**Table 2:** **Plasmids used in this study**.

pUT18	Karimova et al., 1998
pUT18-MmpL7	Gannoun-Zaki et al., 2013
pUT18-NarI	This study
pUT18-NarK2	This study
pUT18-Rv2617c	Klepp et al., 2009
pUT18-SecG	This study
pKT25-KdpF	Gannoun-Zaki et al., 2013
pKT25-KdpFΔNter	This study
pMV261	Stover et al., 1991
pKdpF	Gannoun-Zaki et al., 2013

### Preparation of bacterial lysates and western blot analysis

*Escherichia coli* BTH101 strain transformed with plasmid encoding MmpL7-T18, NarI-T18, or Rv2617c-T18 fusions was grown overnight at 30°C in 250 μl of LB Broth containing 100 μg/ml ampicillin, 0.5 mM IPTG and 70 μM of synthetic KdpF peptide. A control experiment was performed using *E. coli* BTH101 carrying the SecG-T18 plasmid, a membrane protein that does not interact with KdpF peptide. Bacterial cultures were centrifuged, resuspended in 50 μl Laemmli buffer and incubated for 5 min at 95°C. Bacterial lysates were loaded on 12.5% SDS-PAGE and transferred onto nitrocellulose membrane (Invitrogen) for immunoblotting. Mouse anti-T18 antibodies (monoclonal antibody sc-13582, Santa-Cruz) was used at 1:1000 dilution. Mouse anti-DnaK antibody (Tebubio) at 1:5000 dilution was used as loading control. Secondary antibodies labeled with IRDye 800 CW infrared dyes (LICOR) were used and the images were acquired on LICOR Odyssey Fc Imaging System to quantify the amount of proteins.

Dose-effect experiment was similarly conducted with various KdpF synthetic peptide concentrations (14, 35, 70, and 140 μM, respectively).

### Statistical analysis

Statistical analyses were performed by using an unpaired Student *t*-test. Only *P* < 0.05 were considered as statistically significant.

## Results

### KdpF overproducing strain is more susceptible to exogenous NO and accumulates more NO in infected macrophages

**Figure 1:**
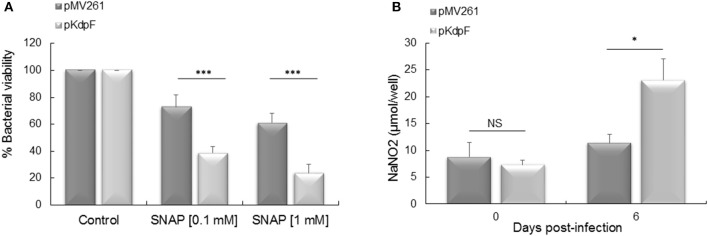
**Mycobacterial susceptibility to exogenous NO and NO accumulation in infected macrophages. (A)** Bacteria were grown in Sauton's medium containing 0.1 mM or 1 mM SNAP for 5 h at 37°C. The *M. bovis* BCG viability was determined by CFUs counts. The results represent the percentage of viability related to non-treated bacteria and are expressed as the mean ± *SD* of four independent experiments (each performed in duplicate). *P*-values were determined by the Student *t-*test (^***^P < 0.001). **(B)** Quantification of NaNO_2_ produced by THP-1 macrophages infected with *M. bovis* BCG-pMV261 and *M. bovis* BCG-pKdpF. ${\text{NO}}_{2}^{-}$ production by macrophages at day 0 (3 h) and day 6 post-infection was quantified using the Griess reagent. Results are expressed as means ± *SD* of three independent experiments and the student's *t*-test was used to determine the statistical significance (^*^P < 0.05).

In a previous study, we showed that replication inside human macrophages of *M. bovis* BCG overproducing KdpF was significantly reduced in comparison to the *M. bovis* BCG-pMV261 control strain (Gannoun-Zaki et al., 2013). In order to determine whether the NO could be responsible for the impaired intracellular multiplication of KdpF overproducing bacteria, we first determined the susceptibility of bacterial strains to NO and then measured its production in infected THP-1 macrophages. For this purpose, *M. bovis* BCG-pMV261 and *M. bovis* BCG-pKdpF strains were treated for 5 h with 0.1 and 1 mM SNAP, a compound releasing NO (Ferrero et al., 1999). The addition of 0.1 and 1 mM SNAP in *M. bovis* BCG-pMV261 bacteria led to a slight diminution of bacterial viability (73 and 61%, respectively). Notably, the effect of SNAP on viability was significantly more pronounced for *M. bovis* BCG-pKdpF strain (39 and 24% for 0.1 and 1 mM SNAP-treated bacteria, respectively) (Figure 1A). This shows that KdpF-overproducing bacteria are more susceptible to nitrosative stress than bacteria carrying the pMV261 control vector.

We next quantified the amount of NaNO_2_ released from infected macrophages into the culture medium using the Griess reagent. A significant higher amount of NaNO_2_ was obtained in macrophages infected with the KdpF overproducing strain as compared to the *M. bovis* BCG-pMV261 infected cells after 6 days of infection (23.12 vs. 11.38 μmol NaNO_2_/well), (Figure 1B). This indicates that macrophages infected with KdpF overproducing bacteria accumulate more ${\text{NO}}_{2}^{-}$ as compared to *M. bovis* BCG-pMV261 infected macrophages. This result suggests that the *M. bovis* BCG-pKdpF strain has hampered NO detoxification.

### Nitrosative stress is involved in the reduced intramacrophage growth of KdpF-overproduced strain

**Figure 2:**
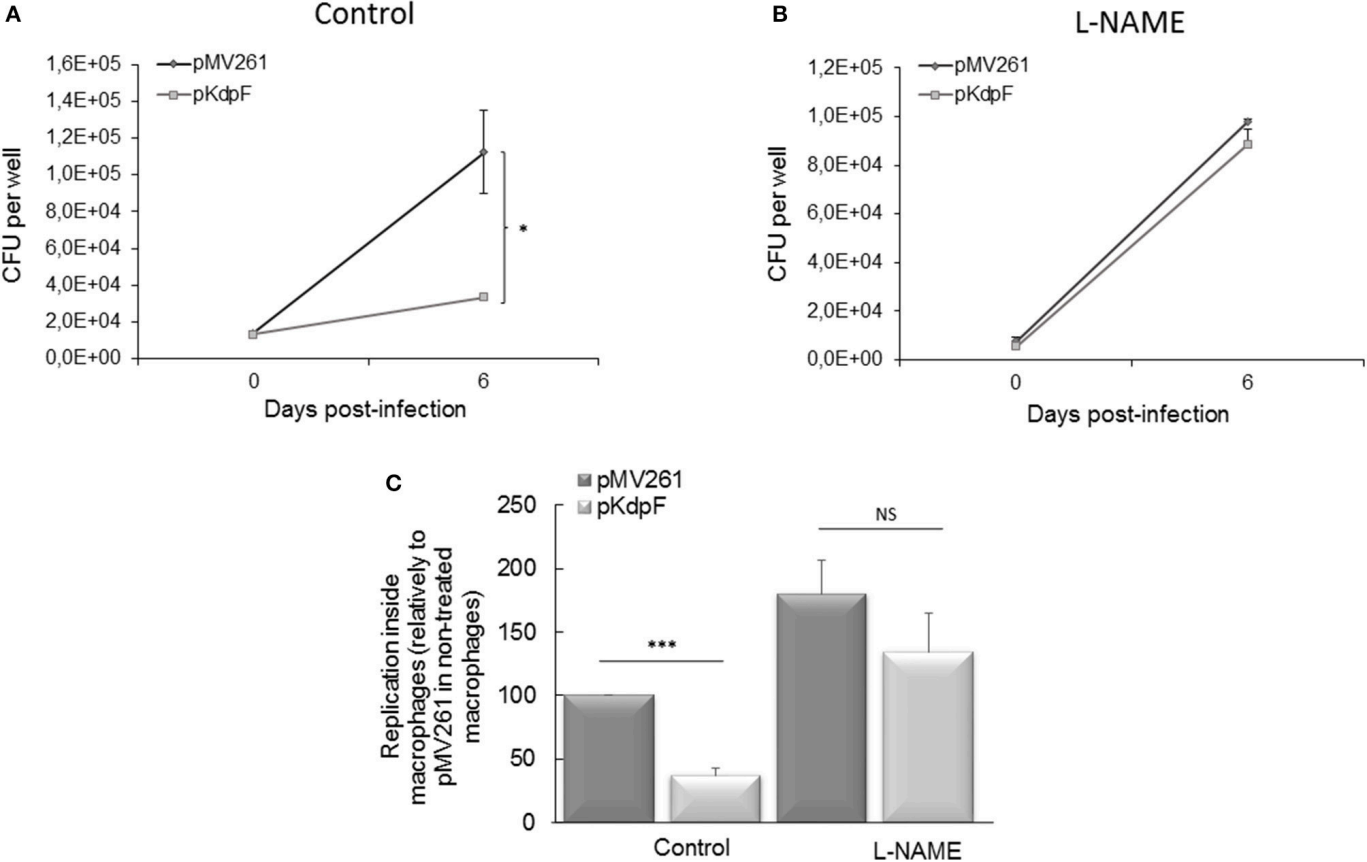
**Mycobacterial intracellular replication in presence of RNIs inhibitor L-NAME. (A)** Human THP-1 macrophages were infected with *M. bovis* BCG-pMV261 and *M. bovis* BCG-pKdpF and lysed after 3 h (day 0) and 6 days. The bacteria replication was determined by CFUs counts. **(B)** Cells were treated with 200 μM L-NAME prior to infection with *M. bovis* BCG-pMV261 and *M. bovis* BCG-pKdpF and lysed as described above. **(C)** Data are presented as percent bacterial replication at 6 days post-infection relative to the 3 h time point for each strain, and reported as 100% for the *M. bovis* BCG-pMV261 strain without L-NAME. Data are the average of three independent experiments. Control viability of *P*-values were determined by the Student's *t*-test (^***^P < 0.001, ^*^P < 0.01).

To evaluate whether the NO accumulation is responsible for the decreased intracellular replication of *M. bovis* BCG-pKdpF strain, infections were carried on human THP1-macrophages pre-treated or not with the reactive nitrogen intermediates (RNIs) inhibitor (L-NAME). In the absence of L-NAME, the strain overproducing KdpF showed a lower replication rate than *M. bovis* BCG-pMV261 strain after 6 days of infection as measured by CFUs counting (Figure 2A), which agrees with our previous results reported with primary human macrophages (Gannoun-Zaki et al., 2013). However, this defect was suppressed upon treatment of THP1- macrophages with L-NAME since similar CFUs counts were obtained after macrophage infection with both strains (Figure 2B). A representation of the multiplication rate (ratio J6/J0), considering non-treated pMV261 strain as 100%, clearly shows that the significant difference between the two strains is suppressed upon SNAP treatment (Figure 2C). Conversely, NO inhibition by L-NAME led to a slight increase of intracellular replication percentage (180% ± 27) of *M. bovis* BCG-pMV261 strain when compared to non-treated infected cells, thus revealing that NO stress limits bacterial intracellular replication. On the other hand, we have considered the ROS-mediated killing as well by using the specific ROS inhibitor NAC (N-Acetyl-L-Cystein) prior to macrophage infection. As shown in Figure S2, no recovery of bacterial replication was obtained in NAC-treated macrophages infected with *M. bovis* BCG overproducing KdpF. Taken together, our results show that RNIs produced upon infection are involved in the reduced replication of the KdpF overproducing strain, suggesting that the increased susceptibility of this strain to nitrosative stress is correlated to the growth defect inside macrophages.

### Effect of KdpF on gene expression during nitrosative stress

**Figure 3:**
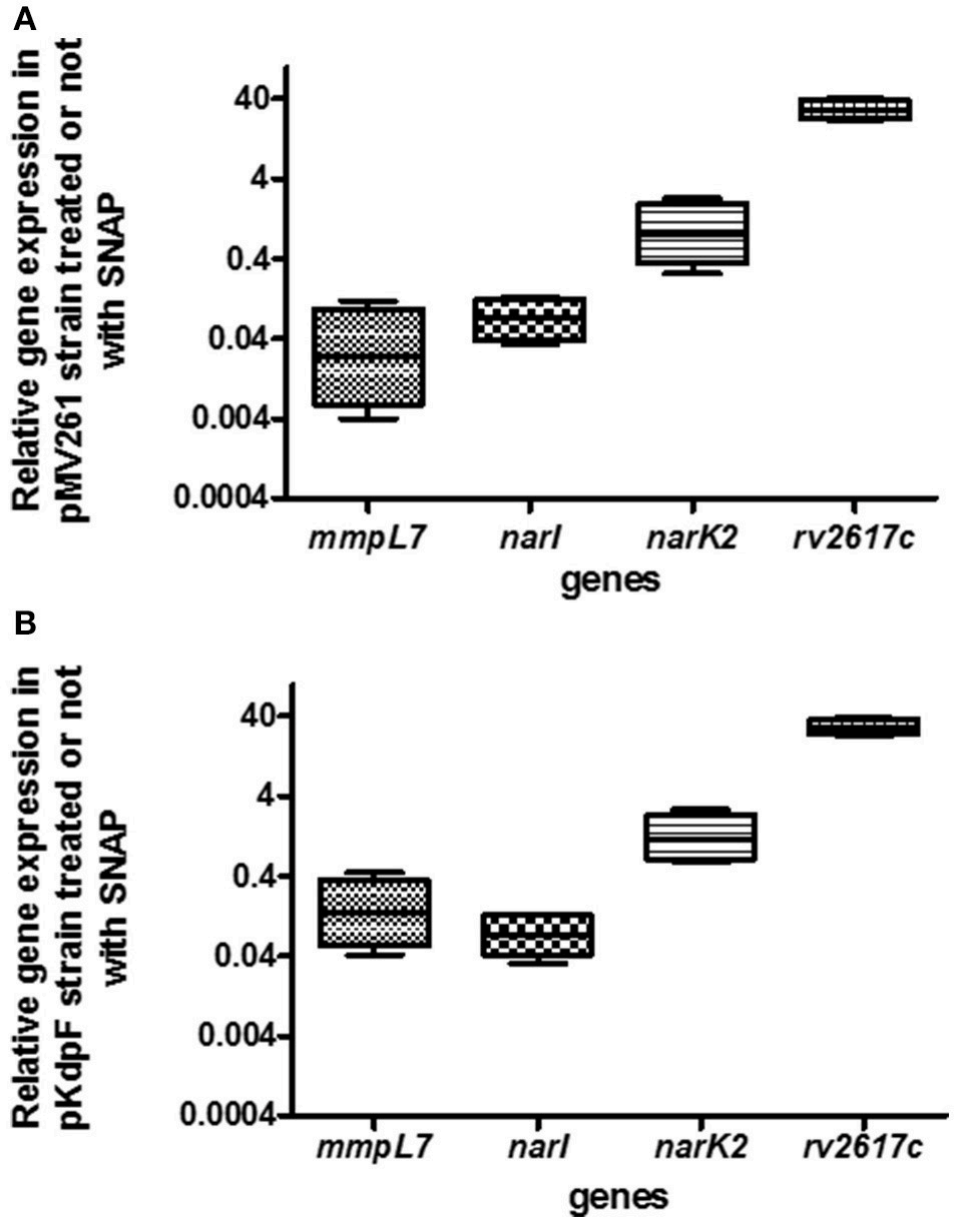
**Quantitative gene expression of *mmpL7*, *narI*, *narK2* and *rv2617c* genes upon exposure to SNAP in control and KdpF overproducing BCG strains. (A)** The *M. bovis* BCG-pMV261 and **(B)** *M. bovis* BCG-pKdpF bacteria were exposed to 1 mM SNAP for 5 h and expression level of specific genes in SNAP-treated samples were evaluated by real-time PCR for each gene and compared to non-treated bacilli. 16S rRNA was used as the internal control for real-time PCR analysis. For each gene, fold induction is expressed as follows: levels of expression relatively to 16S by SNAP-treated *M. bovis* BCG/levels of expression relatively to 16S by non-treated bacilli. Results are analyzed with REST® program and plotted using GraphPad Prism software. Boxes represent the middle 50% of observations. The dotted line represents the median gene expression. Whiskers represent the minimum and maximum observations. Data represents the mean of results derived from real-time PCR analysis of four independent experiments (each performed in triplicate).

Reactive nitrogen intermediates are detoxified through several reactions involving Nar and Nir proteins family to finally produce non-toxic ammonium. The effect of KdpF on nitrosative stress proteins was first investigated at the transcriptional level by quantifying gene expression in both *M. bovis* BCG-pKdpF and *M. bovis* BCG-pMV261 strains. Among the genes involved in the process of NO detoxification, we focused our study on two membrane proteins encoded by *narI* and *narK2* genes. Besides these genes, *rv2617c* and *mmpL7* genes described to be, respectively up- and down-regulated in mycobacteria upon NO stress were used as controls (Ohno et al., 2003; Cossu et al., 2013). For this purpose, quantitative RT-PCR was carried out using RNA extracted from both *M. bovis* BCG strains grown for 5 h in the presence or absence of 1 mM SNAP. Results obtained from the SNAP-treated *M. bovis* BCG-pMV261 strain showed that the expression of the *rv2617c* gene was clearly upregulated by nitrosative stress (Figure 3A), to an extent that was even greater than the one described in *M. tuberculosis* (Ohno et al., 2003). On the other hand, the *nar* genes were not upregulated in SNAP-treated bacteria (Figure 3A), which contrasts with what has been described in *M. tuberculosis* (Cossu et al., 2013). As expected, expression of the *M. bovis* BCG *mmpL7* gene was strongly repressed in presence of SNAP, as previously described for PDIM synthesis genes in *M. tuberculosis* (Cossu et al., 2013). The regulation of *narI, narK2, rv2617c*, and *mmpL7* gene expression upon SNAP treatment was then investigated in the *M. bovis* BCG strain overproducing KdpF (Figure 3B). A similar pattern of expression, as obtained with *M. bovis* BCG-pMV261 strain, was observed, thus indicating that KdpF does not play a role on transcriptional regulation of these genes under nitrosative stress.

### KdpF interacts with membrane proteins related to nitrosative stress response

Membrane peptides have been shown to possess regulatory functions through direct interaction with membrane proteins (Alix and Blanc-Potard, 2009). Since we demonstrated that KdpF had no effect on transcriptional regulation of genes involved in nitrosative stress, we then hypothesized that KdpF could modulate the function or stability of these proteins through direct interaction.

**Figure 4:**
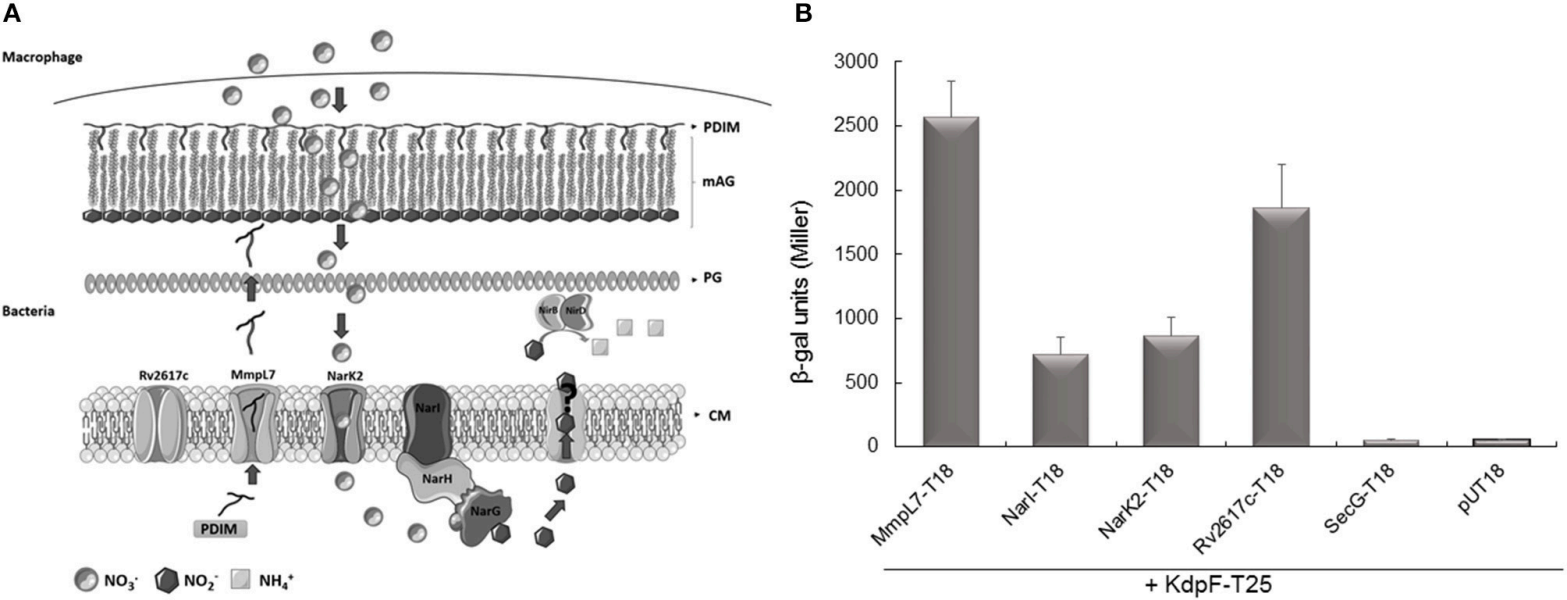
**Interaction of KdpF with membrane proteins related to nitrosative stress. (A)** Membrane localization of MmpL7, NarI, NarK2 and Rv2617c in *M. tuberculosis*. The inorganic source of nitrate (${\text{NO}}_{3}^{-}$) is imported inside the bacteria through the nitrate reductase NarK2 and is reduced to nitrite (${\text{NO}}_{2}^{-}$) by the nitrate reductase complex (NarGHI). Nitrites are further exported by an unknown transporter where there are reduced to ammonium (${\text{NH}}_{4}^{+}$) by the nitrite reductase NirBD. Rv2617c, a transmembrane protein, could be involved in the nitrosative stress since this protein appears to be upregulated when the bacteria are grown in nitrosative stress conditions. On the other hand, the synthesis of PDIM lipids, transported by the MmpL7 transporter, is down-regulated upon nitrosative stress. PDIM (Phthiocerol Dimycocerosate), mAG (Mycolyl-arabinogalactan layer), PG (Peptidoglycan), CM (Cytoplasmic membrane). **(B)** *In vivo* protein interaction with KdpF using the BACTH system. *E. coli* BTH101 strains were co-transformed with plasmids encoding KdpF-T25 fusion proteins and MmpL7-T18, NarI-T18, NarK2-T18, Rv2617c-T18 or SecG-T18, respectively. The basal level of β-galactosidase activity measured with the pUT18 vector is approximately 50 Miller units. Liquid β-galactosidase assays were performed from four independent experiments.

We thus examined the interaction between KdpF and the membrane proteins NarI, NarK2, Rv2617c, and MmpL7 (Figure 4A) using the bacterial two-hybrid (BACTH) system that has been previously validated for detecting interactions between inner-membrane proteins in living bacteria (Karimova et al., 2005). Constructs were made to fuse the T18 subunit of the adenylate cyclase to these diverse membrane proteins (plasmid constructs are described in Table 2) and interaction was tested with the KdpF peptide fused to the T25 subunit (Gannoun-Zaki et al., 2013).

Plasmids encoding the T18 and T25 fusion proteins were then introduced into an *E. coli cya* mutant (BTH101) and functional complementation was determined by measuring β-galactosidase activity (Figure 4B). We have previously shown that the MmpL7 transporter, involved in the PDIM transport highly interacted with KdpF (Gannoun-Zaki et al., 2013), and the MmpL7-T18 construct was thus used as positive control. A high level of β-galactosidase activity (1866 Miller units) was observed with Rv2617c-T18 plasmid, thus indicating a robust interaction between Rv2617c and KdpF. An interaction was also observed between NarI and KdpF, and NarK2 and KdpF (720 and 868 Miller units, respectively). Similar levels of β-galactosidase activity were obtained when reversing the bait and the prey for Rv2617c and NarI (2030 Miller units for assay with Rv2617c-T25 and KdpF-T18 and 1200 Miller units for assay with NarI-T25 and KdpF-T18, data not shown), thus confirming significant interaction. On the other hand, no interaction (β-galactosidase activity similar to the negative control pUT18/KdpF-T25) was detected between KdpF and SecG, a membrane protein unrelated to nitrosative stress used as control. Cumulatively, these results indicate that KdpF peptide can interact significantly and specifically with membrane proteins related to nitrosative stress, which could in turn modulate the regulation of these proteins through protein-protein interaction.

### Effect of the synthetic KdpF peptide on the stability of KdpF partners

**Figure 5:**
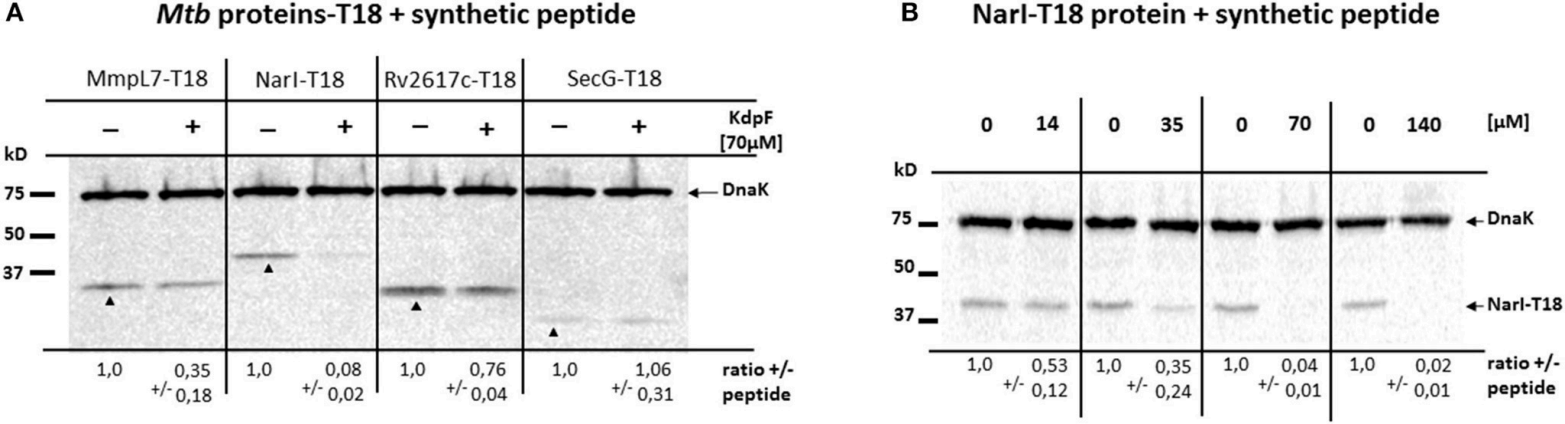
**Effect of synthetic KdpF peptide on protein stability. (A)** *E. coli* BTH101 strain was transformed with plasmids encoding MmpL7-T18, NarI-T18, Rv2617c-T18 or SecG-T18 fusions. KdpF synthetic peptide was added at 70 μM to the cultures and bacterial lysates were loaded on 12.5% SDS-PAGE. The analysis of the amount of T18 fusion proteins was performed by western blotting using mouse anti-T18 antibodies. Mouse anti-DnaK antibodies were used to detect the DnaK protein as internal loading control. **(B)** Dose-response of synthetic KdpF peptide on NarI protein stability. *E.coli* BTH101 strain transformed with NarI-T18 fusion protein was cultured in presence of synthetic KdpF peptide at the indicated concentrations and bacterial lysates were loaded on 12.5% SDS-PAGE and analyzed by western blotting as described above. For both experiments, quantitative data show the mean fold change ± *SD* of each T18 fusion protein, normalized to DnaK in the absence of synthetic peptide. The experiment was repeated three times independently and one representative experiment is shown.

In order to investigate whether the interaction of KdpF peptide with protein partners could interfere with protein stability, we have used a synthetic KdpF peptide that was exogenously added to BTH101 bacteria expressing diverse T18 fusion proteins. While the full-length peptide failed to be synthetized in sufficient soluble amount, a shorter peptide lacking the first 5 amino-acids in its N-terminus was produced (see Materials and Methods). We first validated that this shorter peptide, possessing the same hydrophobic domain as KdpF, carried identical properties in a bacterial two-hybrid assay (Figure S1). Then, the synthetic KdpF peptide was directly added to the culture medium of BTH101 bacteria transformed with various *Mtb*-proteins-T18 derivative plasmids related to nitrosative stress (MmpL7, NarI, NarK2, and Rv2617c). Equal amount of bacterial lysates treated or not with synthetic peptide were analyzed by western blotting using T18-specific antibodies. Our data showed that addition of synthetic peptide (70 μM) resulted in a strong (12-fold) decreased level of NarI-T18 protein, a mild (3-fold) decreased level of MmpL7-T18 protein, whereas only a slight decrease in protein amount was observed for Rv2617c-T18 construct (Figure 5A). On the other hand, no effect was observed upon addition of the synthetic KdpF peptide on the stability of the SecG-T18 control protein. We further performed a dose-response assay with increased concentrations of synthetic KdpF peptide (from 14 to 140 μM) and the NarI-T18 fusion protein (Figure 5B). Our data show a correlation between the increase of KdpF concentration and the decrease of NarI-T18 protein level. Taken together, these results suggest that upon interaction, KdpF peptide could decrease the stability of proteins related to nitrosative stress, mainly NarI and to a lesser extent, MmpL7.

### Synthetic KdpF peptide reduces mycobacterial viability upon NO stress and intramacrophage replication

**Figure 6:**
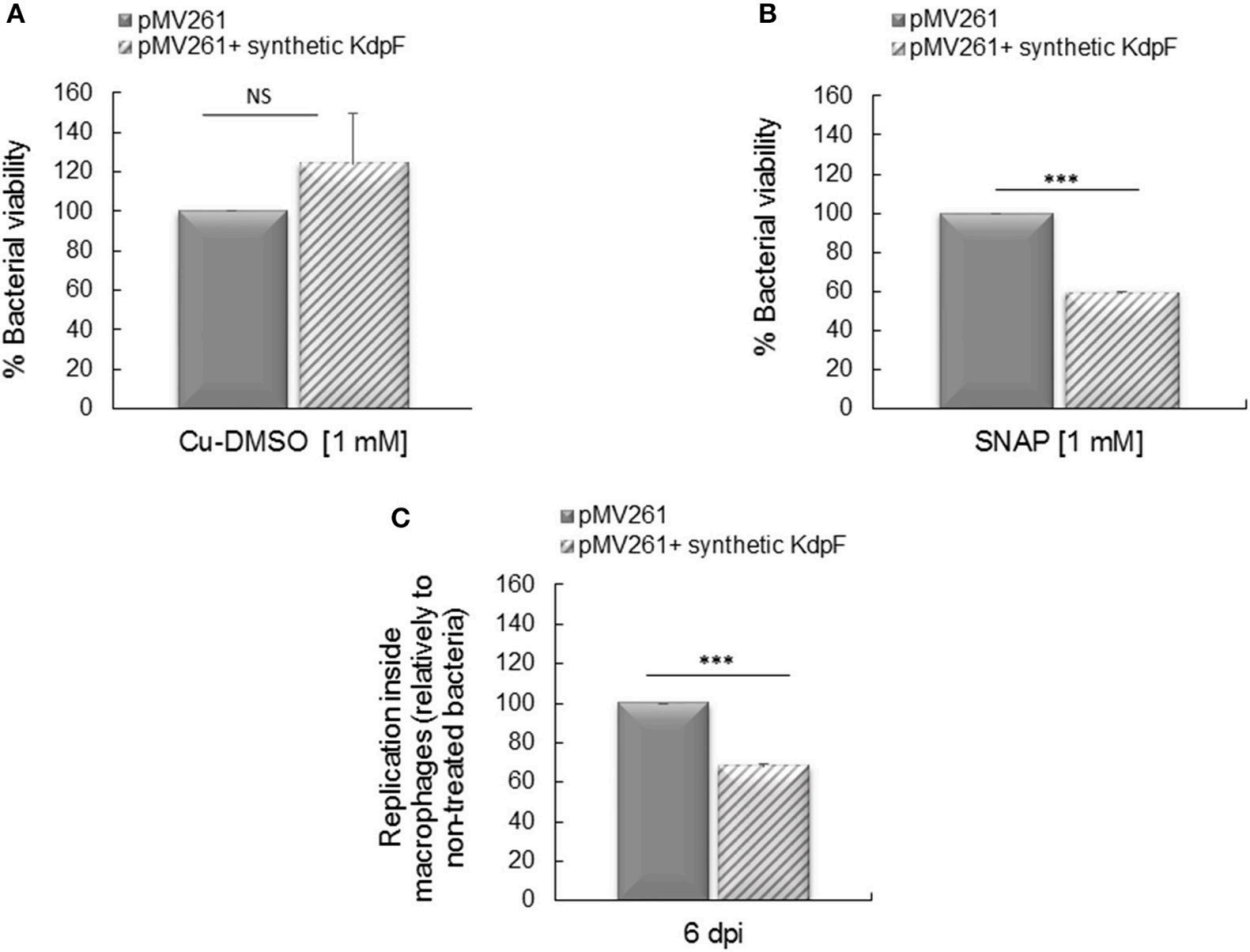
**Effect of synthetic KdpF on bacterial susceptibility to NO and intracellular replication. (A)** Synthetic KdpF peptide (70 μM) was added or not to *M. bovis* BCG-pMV261 culture and bacteria were grown in Sauton's medium or **(B)** bacteria were grown in Sauton's medium supplemented with 1 mM SNAP for 5 h. Bacterial susceptibility was determined by CFUs counts as described. **(C)** Human THP-1 macrophages were infected with *M. bovis* BCG-pMV261 pre-incubated with 70 μM of synthetic KdpF for 10 min. Intracellular replication was determined 6 days post-infection by CFUs counting. The experiments were performed in triplicate and the data was obtained from three independent experiments. Statistical significance was denoted as mean ± *SD* (^***^P < 0.001).

In order to assess the biological activity of exogenously added KdpF peptide on mycobacteria, *M. bovis* BCG-pMV261 strain was first pre-incubated or not with 70 μM of synthetic KdpF peptide, and grown in culture media supplemented or not with the NO donor SNAP. No effect of the exogenous KdpF peptide was observed on bacterial viability when bacteria were grown in the control culture media containing copper, DMSO (Figure 6A). However, when the strain was grown in presence of 1 mM SNAP, a significant diminution of bacterial viability (59%) was measured when bacteria were preloaded with synthetic peptide (Figure 6B). These results suggest that the addition of synthetic KdpF peptide to *M. bovis* BCG-pMV261 bacteria increases the susceptibility of mycobacteria to nitrosative stress, thus mimicking the effect of endogenous KdpF overproduction.

We next investigated the effect of exogenous KdpF peptide addition on mycobacterial intramacrophage replication. The *M. bovis* BCG-pMV261 strain was incubated with 70 μM of synthetic peptide prior to THP-1 macrophage infection. Bacterial multiplication, evaluated by CFUs counts after 6 days of infection, was significantly decreased (68% of multiplication) in bacteria pre-incubated with the exogenous peptide in comparison to the *M. bovis* BCG-pMV261 control bacteria (Figure 6C). Therefore, these results indicate that the exogenous synthetic KdpF peptide impairs intracellular multiplication, again mimicking the effect of endogenous KdpF overproduction, and thus confirming its role as a potent molecule with anti-virulence properties.

## Discussion

The present study aimed to extend our previous work, which demonstrated that the intramacrophage multiplication rate of *M. bovis* BCG overproducing the KdpF peptide was significantly reduced as compared to the *M. bovis* BCG strain carrying the control vector (Gannoun-Zaki et al., 2013). The purpose of this study is to decipher the physiological basis of this phenotype and address the biological effects of exogenously added synthetic peptide.

Mycobacteria are highly adapted to macrophages and possess multiple mechanisms to resist to host immune responses (Ehrt and Schnappinger, 2009). To decipher the role of KdpF in intracellular replication of mycobacteria during macrophage infection, we explored whether this peptide could affect susceptibility of *M. bovis* BCG to nitrosative stress. We demonstrated that overproduction of the endogenous KdpF peptide increased the susceptibility of *M. bovis* BCG to nitrosative stress. Moreover, a higher amount of NO was measured in macrophages infected with the *M. bovis* BCG strain overproducing KdpF as compared to the *M. bovis* BCG-pMV261 strain. The accumulation of NO observed in macrophages infected with *M. bovis* BCG overproducing KdpF could arise from a higher production of NO by iNOS enzyme, from an increased transport of nitrates through the NarK2 transporter or from a lower activity of nitrate reduction by the nitrate reductase complex NarGHIJ (Figure 4A). Furthermore, the growth defect of *M. bovis* BCG bacteria overproducing KdpF was completely relieved upon treatment of macrophages with an inhibitor of nitrosative stress, supporting a causative link between the nitrosative stress sensitivity of the strain and its lower replication within macrophages.

To further evaluate the anti-virulence properties of the KdpF peptide, we conducted for the first time, experiments with exogenous peptide. Interestingly, we showed that incubation of *M. bovis* BCG-pMV261 strain with the synthetic KdpF peptide resulted in susceptibility to nitrosative stress as obtained with the endogenous overproduction of KdpF. It is noteworthy that addition of the synthetic peptide to *M. bovis* BCG-pMV261 bacteria prior to THP-1 infection led to a reduced intramacrophage bacterial replication, again mimicking the effect of endogenous KdpF overproduction. This result provides a proof of concept for an anti-virulence effect mediated by addition of a hydrophobic synthetic peptide. To propose a biological role, we suppose that the exogenous KdpF peptide interacts with the bacteria and inserts into the membrane, thus leading to a decreased resistance to nitrosative stress and intracellular replication. Pretreatment with the peptide before incubation with macrophages does not represent a true clinical model and our next goal will be to carry out experiments with peptide added after phagocytosis (peptide would need to cross the macrophage cell membrane, the phagosomal membrane before reaching the mycobacterial envelope).

To address the mechanism underlying the anti-virulence properties of the KdpF peptide, we have investigated a potential regulatory effect of KdpF on membrane proteins related to nitrosative stress. We first evaluated a potential effect of KdpF peptide on transcriptional regulation of genes related to nitrosative stress. We started by measuring the effect of NO donor (SNAP) on expression level of genes of interest in *M. bovis* BCG, which is less documented than *M. tuberculosis*. Strikingly, *narK2* and *narI* genes were not up-regulated in *M. bovis* BCG upon treatment with NO donor, whereas they are induced in *M. tuberculosis* in similar conditions (Ohno et al., 2003; Cossu et al., 2013). This result is likely due to polymorphisms in the promoter region of *narG* that have been shown to prevent the up-regulation of *nar* genes by NO donor in *M. bovis* BCG (Stermann et al., 2004; Huang et al., 2015). In addition, *narK2* gene is poorly expressed in *M. bovis* and *M. bovis* BCG (Honaker et al., 2008) due to a single-nucleotide polymorphism in the *narK2X* promoter region leading to a reduced promoter activity (Chauhan et al., 2010). In contrast to the *nar* genes, the *rv2617c* gene, was highly induced by SNAP in *M. bovis* BCG, to an extent even greater than the one reported in *M. tuberculosis* (Ohno et al., 2003). Rv2617c protein is a membrane protein, specific to the *M. tuberculosis* complex which can interact with the Erp protein (Klepp et al., 2009). The function of Rv2617c is unknown but the regulation of its gene expression suggests that it plays a role toward nitrosative stress adaptation. In addition, our results showed that *M. bovis* BCG *mmpL7* gene, coding for the transporter of PDIM, was strongly down-regulated by SNAP. This finding is consistent with the fact that PDIM synthesis is strongly repressed by nitrosative stress in *M. tuberculosis*, suggesting a link between PDIM and nitrosative stress (Cossu et al., 2013). The genes tested (*mmpL7, narI, narK2*, and *rv2617c*) were similarly regulated by SNAP in the KdpF overproducing strain comparatively to the *M. bovis* BCG-pMV261 strain, indicating that the KdpF effect is not linked to transcriptional regulation of these genes.

The absence of effect of KdpF peptide at the transcriptional level prompted us to investigate a potential regulatory role directly on proteins related to nitrosative stress. We first addressed the interaction between KdpF and selected membrane proteins (NarI, NarK2, MmpL7, and Rv2617c) using the heterologous *E. coli* bacterial double hybrid system (BACTH). We then took advantage of the diverse recombinant proteins expressed in this system to investigate the effect of KdpF synthetic peptide on protein stability. Our results confirmed the strong interaction of KdpF with MmpL7 (Gannoun-Zaki et al., 2013) and showed that the KdpF peptide was also able to interact more or less strongly with NarI, NarK2, and Rv2617c proteins. No interaction was observed with SecG, a membrane protein unrelated to nitrosative stress, indicative of a specificity in the other interactions. Interestingly, in the presence of exogenous KdpF peptide, we observed a remarkable decrease in NarI protein amount, and to a lesser extend in MmpL7 protein amount, whereas no major degradation could be detected for Rv2617c or SecG proteins. One could conclude that the synthetic KdpF peptide is able to alter protein stability, especially for NarI and MmpL7, through its interaction with these membrane proteins. Although protein interaction assays and protein stability experiments were performed in a heterologous bacterial system, one can speculate that the KdpF peptide may play a regulatory role by interacting with different membrane proteins, specifically NarI and MmpL7 and in turn promoting their degradation. Although the *narGHIJ* genes are weakly expressed in *M. bovis* BCG, they are up-regulated after macrophage infection with mycobacteria (Jung et al., 2013). One could further hypothesize that the susceptibility of the KdpF overproducing bacteria to nitrosative stress is due to the degradation of NarI, thus leading to an accumulation of nitrites inside the bacteria and a reduced intramacrophage replication. It would be of interest to confirm the degradation of NarI in *M. bovis* BCG strain overproducing KdpF and to investigate whether the interaction between KdpF and NarI also occurs under native conditions.

In addition, or alternatively, the degradation of the MmpL7 transporter in *M. bovis* BCG overproducing KdpF could contribute to the increased susceptibility to NO stress by reducing the amount of PDIM at the mycobacterial membrane. In agreement, several evidences suggest that the PDIM contribute to *M. tuberculosis* resistance to antimicrobial agents produced by macrophages against virulent mycobacteria (Chan and Flynn, 2002), including resistance to the nitric oxide-dependent killing of macrophages (Rousseau et al., 2004). Moreover, the PDIM lipids have been recently implicated in host immune evasion by masking pathogen–associated molecule patterns (PAMPs), and the absence of PDIMs promoted the recruitment of macrophages producing reactive nitrogen species (Cambier et al., 2014). However, another study showed that the attenuation of PDIM-deficient *M. tuberculosis* strain is RNIs independent (Day et al., 2014), indicating some controversy in the role of PDIM in the protection of mycobacteria from host-mediated killing.

Our current work showed that overproducing KdpF peptide in *M. bovis* BCG led to an intracellular replication decrease linked to nitrogen detoxification. Interestingly, in *E. coli*, a link between the transport of nitrogen and K^+^ has been found. The nitrogen transport by phosphotransfer system (Ntr-PTS) involved in nitrogen regulation, modulates expression of *kdpFABC* operon. In this system, the dephosphorylated protein IIA-^Ntr^ interacts specifically with the sensor kinase KdpD, stimulating the kinase activity of KdpD which in turn increases *kdpFABC* expression (Lüttmann et al., 2009). Since our previous work indicated that KdpF overproduction did not affect *kdpFABC* operon expression, it should not modulate KdpD kinase activity and it is therefore unlikely that such an Ntr-PTS system regulation is involved in the decrease of intracellular bacterial replication.

In conclusion, our present work demonstrated a causative link between high KdpF level, resulting from endogenous overproduction or from exogenous synthetic peptide, and sensitivity to nitrosative stress, which is turn reduces intramacrophage replication. Our data suggest that the KdpF peptide, by interacting with several proteins involved in nitrosative stress, could promote their degradation, leading to a decrease intracellular multiplication. The biological activity of the synthetic peptide supports our postulate that KdpF could represent a basis for a future anti-virulence molecule in combination with classical antibiotic treatment to circumvent the problem of widespread antibiotic resistance.

## Author contributions

LG-Z, MR-O, and AB-P designed the study. LG-Z and MR-O performed experiments. EV synthesized the peptide. LG-Z, MR-O, and AB-P analyzed the data. LG-Z, AB-P, and MR-O drafted the manuscript. VM contributed to critical review of the manuscript. All authors contributed to read and approved the final version.

### Conflict of interest statement

The authors declare that the research was conducted in the absence of any commercial or financial relationships that could be construed as a potential conflict of interest.

## Supplementary material

Figure S1 ***In vivo* protein interaction of KdpF-ΔNter with MmpL7 using the BACTH system**. *E. coli* BTH101 strains were co-transformed with plasmids encoding the KdpF lacking the first 5 amino-acids, KdpF-ΔNter-T25 or the KdpF-T25 with the MmpL7-T18 fusion proteins, respectively. Liquid β-galactosidase assays were performed from four independent experiments.

Figure S2 **Mycobacterial intracellular replication in presence of ROS inhibitor NAC. (A)** Human THP-1 macrophages were infected with *M. bovis* BCG-pMV261 and *M. bovis* BCG-pKdpF and lysed after 3 h (day 0) and 6 days. The bacteria replication was determined by CFUs counts. **(B)** Cells were treated with 2 mM NAC (N-Acetyl-L-Cystein) prior to infection with *M. bovis* BCG-pMV261 and *M. bovis* BCG-pKdpF and lysed as described above. Data are the average of three independent experiments. Control viability of *P*-values were determined by the Student's *t*-test (^***^*P* < 0.001, ^*^*P* < 0.01).
