# Supplementary figures and images for: Endogenous and Exogenous KdpF Peptide Increases Susceptibility of Mycobacterium bovis BCG to Nitrosative Stress and Reduces Intramacrophage Replication
Source: Front Cell Infect Microbiol. 2017 Apr 6;7:115. doi: 10.3389/fcimb.2017.00115 (PMC5382158; doi:10.3389/fcimb.2017.00115)

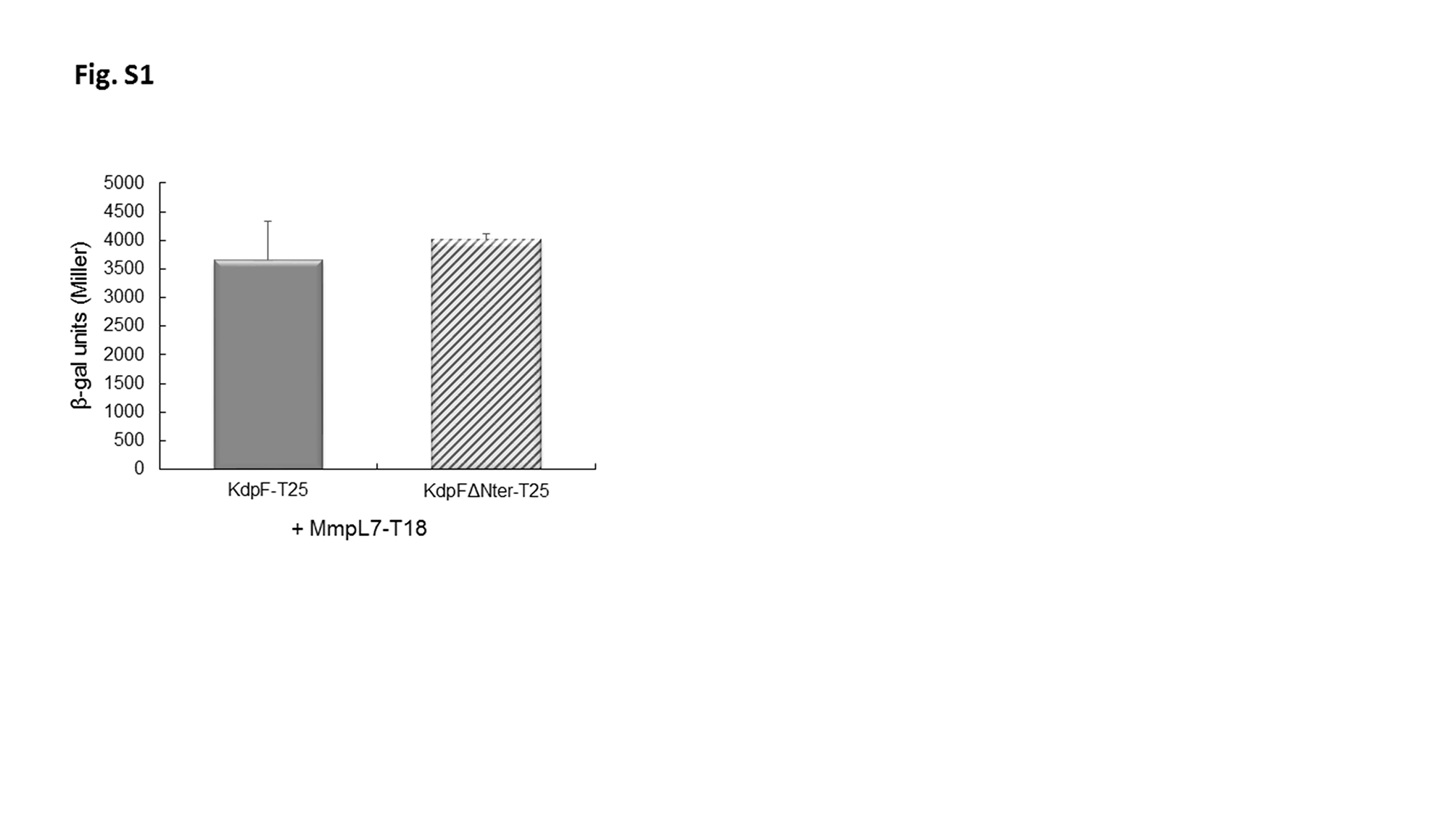

Supplement: Figure S1 — In vivo protein interaction of KdpF-ΔNter with MmpL7 using the BACTH system. E. coli BTH101 strains were co-transformed with plasmids encoding the KdpF lacking the first 5 amino-acids, KdpF-ΔNter-T25 or the KdpF-T25 with the MmpL7-T18 fusion proteins, respectively. Liquid β-galactosidase assays were performed from four independent experiments. [file Image1.TIF]

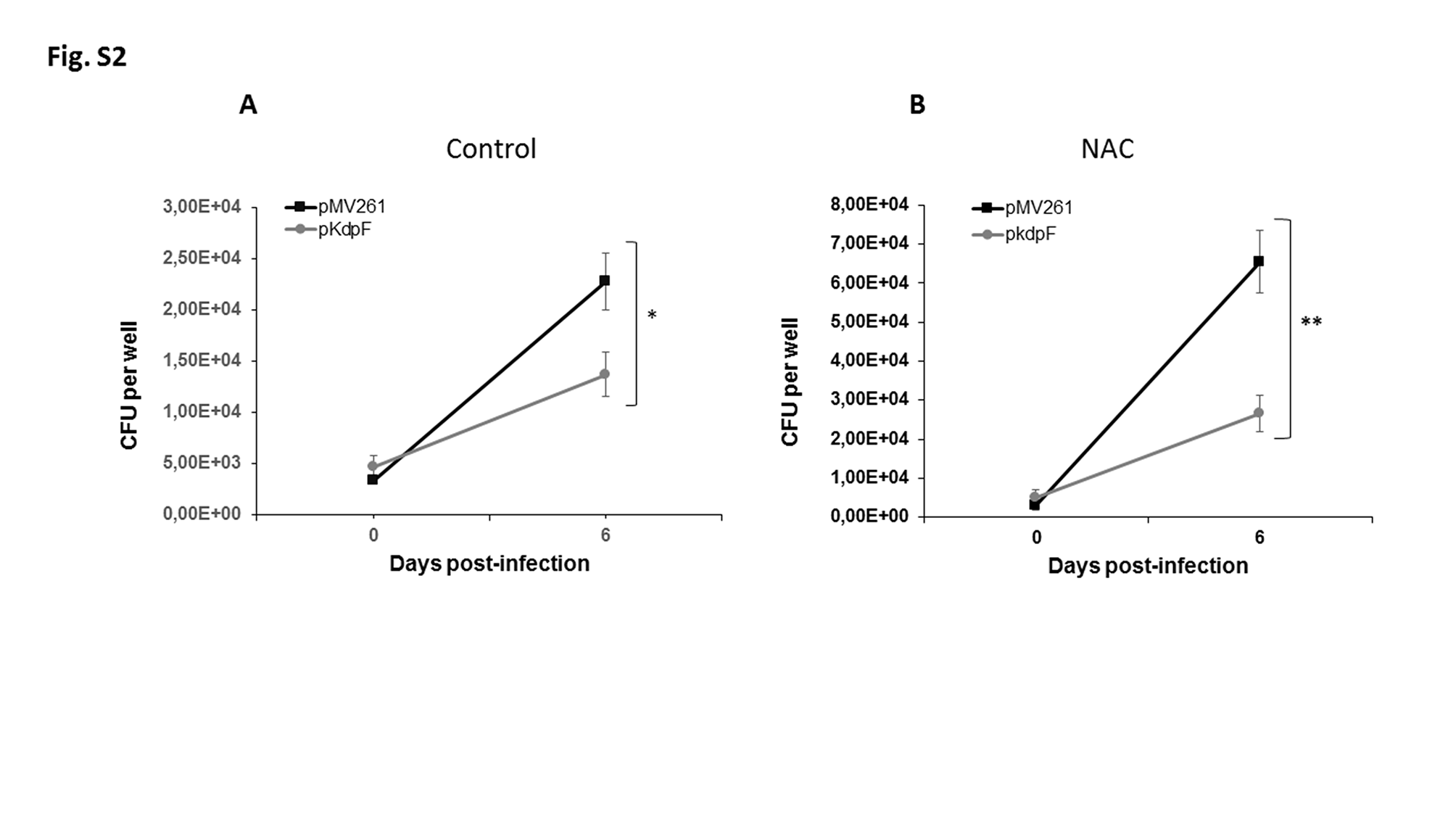

Supplement: Figure S2 — Mycobacterial intracellular replication in presence of ROS inhibitor NAC. (A) Human THP-1 macrophages were infected with M. bovis BCG-pMV261 and M. bovis BCG-pKdpF and lysed after 3 h (day 0) and 6 days. The bacteria replication was determined by CFUs counts. (B) Cells were treated with 2 mM NAC (N-Acetyl-L-Cystein) prior to infection with M. bovis BCG-pMV261 and M. bovis BCG-pKdpF and lysed as described above. Data are the average of three independent experiments. Control viability of P-values were determined by the Student's t-test (***P < 0.001, *P < 0.01). [file Image2.TIF]
